# Supplementary material for: Self-collection and pooling of samples as resources-saving strategies for RT-PCR-based SARS-CoV-2 surveillance, the example of travelers in French Polynesia
Source: PLoS One. 2021 Sep 2;16(9):e0256877. doi: 10.1371/journal.pone.0256877 (PMC8412272; doi:10.1371/journal.pone.0256877)
Supplement: S1 File — (PDF) [file pone.0256877.s001.pdf]

COVID-19: INFORMATION FOR TRAVELERS ENTERING FRENCH POLYNESIA

Dear traveler,

Welcome to French Polynesia.

In order to protect our country from the introduction of coronavirus SARS-CoV-2, a mandatory health surveillance system for the travelers has been implemented by the country (Arrêté n° 525 CM of May 13, 2020).

Before your departure, you were asked to provide a negative test for SARS-CoV-2 performed during the 3 days preceding the flight, and to fill out a digital health commitment form on the website www.etis.pf. The information collected on this form should enable to retrace your itinerary if you develop the disease during your stay in French Polynesia.

As the test performed before your departure does not completely exclude the risk that you are infected by SARS-CoV-2, **another test (free of charge) will be carried out during the week following your arrival in French Polynesia, for all travelers aged at least 6 years old.**
This test consists in an oral and nasal swab performed by yourself, at the date* indicated on the envelope containing the kit that you are issued upon arrival in French Polynesia (see self-sampling instructions).

- At the date of the test, if you are staying in any accommodation or cruise ship participating in the surveillance of travelers, as soon as the sample collection is performed, you drop it at the reception of the establishment that will be responsible for the delivery to a health care center as listed in the appendix.
- Otherwise, you will quickly drop your sample at a health care center or directly at the Institut Louis Malardé in Papeete, at opening hours indicated in the appendix.

**If you are staying on an island served less than 3 times a week by plane, please contact the island's health care center to adjust the collection date according to the next flight.*

This test being carried out as part of a surveillance system, the result will not be communicated to you. However, if a SARS-CoV-2 infection is suspected, you will be contacted by the Health surveillance office of the Direction de la santé.

Reminders:

If during your stay you have at least one of the symptoms of Covid-19 (fever, cough, sore throat, headache, diarrhea, breathing difficulties, body aches, loss of taste or smell), contact immediately the **reporting platform** at the following number: **(+689) 40 455 000**. If you have sign s of severity, call directly the **SAMU at (+689) 15**.

Throughout your stay in French Polynesia, apply scrupulously the **barrier measures**: respect for physical distance of at least 1 meter, wearing a mask in public areas, and regular cleaning of the hands with soap or hand disinfection with a hydro-alcoholic solution.

For any information , please contact the Institut Louis Malardé every day from 8am to 4 pm
phone: (+689) 40 416 459 / e-mail: Cov-check@ilm.pf

Your collaboration is essential to preserve the health of the population of French Polynesia.

Thank you for your cooperation

COVID-19 : SURVEILLANCE OF TRAVELERS IN FRENCH POLYNESIA
SELF-SAMPLING INSTRUCTIONS

Sampling kit components

- « Biohazard » bag
- Absorbant (leave into the bag)
- Swab for oral sampling
- Swab for nasal sampling
- Tube with transport liquid inside
- « Barcode » bag

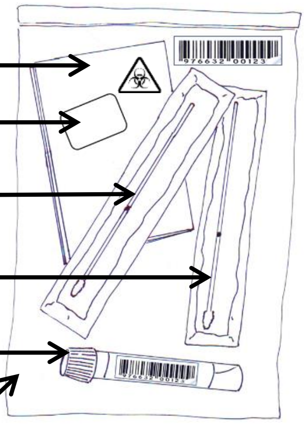

Instructions for the nasal sampling

Make sure not to eat, drink, smoke or brush your teeth at least 20 minutes before the sampling.

1. Blow your nose with a clean tissue.
2. Wash your hands (with soap or hydro-alcoholic solution).
3. Open the tube containing the transport liquid.
4. Place this tube in a small, stable container (glass, cup) to avoid spilling it.
5. Remove the swab for the nasal sampling from the wrapper by holding the plastic handle. **Do not touch the cotton tip!**
6. Insert the entire cotton tip of the swab into one nostril until you feel a bit of resistance, then rub it in a circle around your nostril 4 times. Repeat the previous action in the second nostril using the same swab.
7. Break the swab at the break point. **Do not touch the cotton tip!**
8. Put the cotton end of the swab in the tube containing the transport liquid (the cotton end must be immersed in the liquid).

Instructions for the oral sampling

9. Take a deep breath then cough 5 times in your elbow.
10. Remove the swab for the oral sampling from the wrapper by holding the plastic handle. **Do not touch the cotton tip!**
11. Insert the cotton tip of the swab into the mouth. Rub several times inside of both cheeks, the top and the bottom of the tongue, the palate, and finally the lower and upper gums (make sure the cotton is well soaked in saliva).
12. Break the swab at the break point. **Do not touch the cotton tip!**
13. Put the cotton end of the swab **in the tube containing the transport liquid previously used for the nasal sampling.**
14. Close the tube by firmly screwing the cap.
15. Turn the tube 3 times to mix the samples impregnated on the cotton with the liquid.
16. Put the tube in the « biohazard » bag then make sure to close the bag tightly (do not remove the absorbant).
17. Put the « biohazard » bag in the « barcode » bag and seal it (do net remove the barcodes stuck on the bag). Store the bag in the refrigerator (between 4°C and 8°C).
18. Give the bag to the referent of your accommodation or drop it in a health care center (see the list provided in the appendix).

For any information , please contact the Institut Louis Malardé every day from 8am to 4 pm
phone: (+689) 40 416 459 / e-mail: Cov-check@ilm.pf

List of health care centers :

➢ Tahiti

•Institut Louis Malardé, Papeete – Tel: (+689) 40 416 459. Open from Monday to Friday from 6.30am to 4pm and Saturday from 8am to 12pm
•Dispensaire de Papeete – Tel: (+689) 40 549 838. Open from Monday to Thursday 7am to 3pm, Friday from 7am to 2pm
•Dispensaire de Faa'a – Tel: (+689) 40 850 857. Open from Monday to Thursday 7am to 3pm, Friday from 7am to 2pm
•Dispensaire de Punaauia – Tel (+689) 40 503 770. Open from Monday to Thursday 7am to 3pm, Friday from 7am to 2pm
•Dispensaire de Paea – Tel: (+689) 40 533 259. Open from Monday to Thursday 7am to 3pm, Friday from 7am to 2pm
•Dispensaire de Papara – Tel: (+689) 40 574 787. Open from Monday to Thursday 7am to 3pm, Friday from 7am to 2pm
•Dispensaire de Teva I Uta – Tel: (+689) 40 547 270. Open from Monday to Thursday 7am to 3pm, Friday from 7am to 2pm
•Dispensaire de Arue – Tel: (+689) 40 455 959. Open from Monday to Thursday 7am to 3pm, Friday from 7am to 2pm
•Dispensaire de Mahina – Tel: (+689) 40 481 885. Open from Monday to Thursday 7am to 3pm, Friday from 7am to 2pm
•Dispensaire de Papenoo – Tel: (+689) 40 423 671. Open from Monday to Thursday 7am to 3pm, Friday from 7am to 2pm
•Dispensaire de Tiarei – Tel: (+689) 40 521 633. Open from Monday to Thursday 7am to 3pm, Friday from 7am to 2pm
•Dispensaire de Hiti'aa – Tel: (+689) 40 521 019. Open from Monday to Thursday 7am to 3pm, Friday from 7am to 2pm
•Hôpital de Taravao – Tel: (+689) 40 547 782. Open from Monday to Thursday 7:30am to 3:30pm, Friday from 7:30am to 2:30pm

➢Moorea

•Hôpital d'Afareaitu – Tel: (+689) 40 552 222. Open from Monday to Thursday 7am to 3pm, Friday from 7am to 2pm

➢ Raiatea

•Hôpital d'Uturoa – Tel: (+689) 40 600 800. Open from Monday to Thursday 7:30am to 3:30pm, Friday from 7:30am to 2:30pm

➢ Tahaa

•Centre médical – Tel: (+689) 40 656 331. Open from Monday to Thursday 7:30am to 3:30pm, Friday from 7:30am to 2:30pm
•Centre médical – Tel: (+689) 40 656 751. Open on Tuesday from 7:30am to 12:00am

➢Huahine

•Dispensaire – Tel: (+689) 40 688 248. Open from Monday to Thursday 7:30am to 3:30pm, Friday from 7:30am to 2:30pm

➢Bora Bora

•Dispensaire – Tel: (+689) 40 677 077. Open from Monday to Thursday 7:30am to 3:30pm, Friday from 7:30am to 2:30pm

➢Maupiti

•Infirmerie – Tel: (+689) 40 678 018. Open from Monday to Thursday 7:30am to 3:30pm, Friday from 7:30am to 2:30pm

➢Nuku Hiva

•Hôpital Taiohae – Tel: (+689) 40 910 200. Open from Monday to Thursday 7:30am to 3:30pm, Friday from 7:30am to 2:30pm
•Infirmerie de Taipivai – Tel: (+689) 40 920 142. Open from Monday to Friday 7:30am to 1:30pm
•Infirmerie de Hatiheu – Tel: (+689) 40 920 143. Open from Monday to Friday 7:30am to 1:30pm

➢Ua Huka

•Infirmerie de Hane – Tel: (+689) 40 926 058. Open from Monday to Friday 7:30am to 1:30pm

➢Ua Pou

•Centre médical de Hakahau – Tel: (+689) 40 925 375. Open from Monday to Thursday 7:30am to 3:30pm, Friday from 7:30am to 2:30pm
•Infirmerie de Hakamaii – Tel: (+689) 40 925 299. Open from Monday to Friday 7:30am to 1:30pm
•Infirmerie de Hakatao – Tel: (+689) 40 925 104. Open from Monday to Friday 7:30am to 1:30pm

➢Hiva Oa

•Centre médical de Atuona – Tel: (+689) 40 927 375. Open from Monday to Thursday 7:30am to 3:30pm, Friday from 7:30am to 2:30pm
•Infirmerie de Paumau – Tel: (+689) 40 927 496. Open from Monday to Friday 7:30am to 1:30pm

➢Tahuata

•Infirmerie de Vaitahu – Tel : (+689) 40 929 227. Open from Monday to Friday 7:30am to 1:30pm

➢Fatu Hiva

•Infirmerie de Omoa – Tel: (+689) 40 928 036. Open from Monday to Friday 7:30am to 1:30pm

➢Tubuai

•Centre médical de Mataura – Tel: (+689) 40 932 250. Open from Monday to Thursday 7:30am to 3:30pm, Friday from 7:30am to 2:30pm

➢Rurutu

•Centre médical de Moerai – Tel: (+689) 40 930 440. Open from Monday to Thursday 7:30am to 3:30pm, Friday from 7:30am to 2:30pm

➢Rimatara

•Infirmerie de Amaru – Tel: (+689) 40 944 270. Open from Monday to Thursday 7:30am to 3:30pm, Friday from 7:30am to 2:30pm

➢Raivavae

•Infirmerie de Rairua – Tel: (+689) 40 95 42 31. Open from Monday to Thursday 7:30am to 3:30pm, Friday from 7:30am to 2:30pm

➢Rangiroa

•Centre médical de Avatoru – Tel: (+689) 40 960 325.
•Infirmerie de Tiputa – Tel: (+689) 40 967 396. Open from Monday to Friday 7:30am to 12:00am. Emergency 24h/24, everyday

➢Hao

•Centre médical – Tel: (+689) 40 970 513.

➢Makemo

•Centre médical – Tel: (+689) 40 980 325. Open from Monday to Thursday 7:30am to 3:30pm, Friday from 7:30am to 2:30pm
•Infirmerie – Tel: (+689) 40 980 325. Open from Monday to Friday 7:30am to 12:00am. Emergency 24h/24, everyday

➢**Gambier**

•Centre médical de Rikitea – Tel: (+689) 40 978 216. Open from Monday to Thursday 7:30am to 3:30pm, Friday from 7:30am to 2:30pm

➢**Ahe**

•Infirmerie – Tel: (+689) 40 964 403. Open from Monday to Friday 7:30am to 12:00am. Emergency 24h/24, everyday

➢**Anaa**

•Infirmerie – Tel: (+689) 40 983 204. Open from Monday to Friday 7:30am to 12:00am. Emergency 24h/24, everyday

➢**Arutua**

•Infirmerie – Tel: (+689) 40 965 300. Open from Monday to Friday 7:30am to 12:00am. Emergency 24h/24, everyday

➢**Fakarava**

•Infirmerie – Tel: (+689) 40 984 224. Open from Monday to Friday 7:30am to 12:00am. Emergency 24h/24, everyday

➢**Manihi**

•Infirmerie – Tel: (689) 40 964 136. Open from Monday to Friday 7:30am to 12:00am. Emergency 24h/24, everyday

➢**Napuka**

•Infirmerie – Tel : (+689) 40 973 260. Open from Monday to Friday 7:30am to 12:00am. Emergency 24h/24, everyday

➢**Reao**

•Infirmerie – Tel: (+689) 40 969 041. Open from Monday to Friday 7:30am to 12:00am. Emergency 24h/24, everyday

➢**Takapoto**

•Infirmerie – Tel: (+689) 40 986 486. Open from Monday to Friday 7:30am to 12:00am. Emergency 24h/24, everyday

➢**Takaroa**

•Infirmerie – Tel: (+689) 40 982 263. Open from Monday to Friday 7:30am to 12:00am. Emergency 24h/24, everyday

➢**Tikehau**

•Infirmerie – Tel: (+689) 40 962 349. Open from Monday to Friday 7:30am to 12:00am. Emergency 24h/24, everyday
