# Supplementary material for: Self-collection and pooling of samples as resources-saving strategies for RT-PCR-based SARS-CoV-2 surveillance, the example of travelers in French Polynesia
Source: PLoS One. 2021 Sep 2;16(9):e0256877. doi: 10.1371/journal.pone.0256877 (PMC8412272; doi:10.1371/journal.pone.0256877)
Supplement: S1 Table — (PDF) [file pone.0256877.s002.pdf]

Date of reception at ILM (month/year)	Pooled samples		Individual samples		
	ID	E gene detection (Ct)	ID	E gene detection (Ct)	RdRP gene detection (Ct)
August 2020	Pool 1	16.7	Traveler 1	14.46	16.01
August 2020	Pool 2	27.3	Traveler 2	25.24	26.31
August 2020	Pool 3	26.22	Traveler 3	25.46	25.86
August 2020	Pool 4	32.98	Traveler 4	31.26	31.93
August 2020	Pool 5	28.89	Traveler 5	26.26	26.79
August 2020	Pool 6	27.45	Traveler 6	24.79	26.21
August 2020	Pool 7	29.29	Traveler 7		31.41
August 2020		29.29	Traveler 8		33.18
August 2020		29.29	Traveler 9		29.64
August 2020	Pool 8	17.93	Traveler 10	15.89	16.98
August 2020	Pool 9	35.64	Traveler 11	39.19	39.71
August 2020	Pool 10	15.81	Traveler 12	14.26	15.24
August 2020	Pool 11	31.15	Traveler 13	29.83	29.86
August 2020	Pool 12	20.21	Traveler 14	18.76	17.74
August 2020	Pool 13	18.49	Traveler 15		38.4
August 2020		18.49	Traveler 16		17.76
September 2020	Pool 14	19.26	Traveler 17	17.84	17.02
September 2020	Pool 15	26.29	Traveler 18	ND	24.14
September 2020	Pool 16	32.44	Traveler 19	30.61	30,00
September 2020	Pool 17	37.31	Traveler 20	34.24	32.9
September 2020	Pool 18	34.1	Traveler 21	32.85	31.33
September 2020	Pool 19	20.61	Traveler 22	19.54	19.67
September 2020	Pool 20	21.61	Traveler 23	20.33	19.73
September 2020	Pool 21	24.64	Traveler 24	24.16	22.3
September 2020	Pool 22	21.89	Traveler 25	24.25	22.55
September 2020	Pool 23	27.08	Traveler 26	24.63	24.53
September 2020	Pool 24	33.63	Traveler 27	28.1	24.21
September 2020	Pool 25	25.63	Traveler 28	25.31	25.01
September 2020	Pool 26	28.3	Traveler 29	28.08	25.31
September 2020	Pool 27	27.46	Traveler 30	26,00	25.21
September 2020	Pool 28	22.28	Traveler 31	21.8	20.22
September 2020	Pool 29	18.23	Traveler 32	18.93	18.78
September 2020	Pool 30	20.99	Traveler 33	20.92	19.41
September 2020	Pool 31	25.94	Traveler 34	28.01	26.61
September 2020	Pool 32	21.41	Traveler 35	22.02	20,00
September 2020	Pool 33	25.57	Traveler 36		23.9
September 2020		25.57	Traveler 37		25.6
September 2020	Pool 34	33.5	Traveler 38	29.82	28.25
September 2020	Pool 35	28.9	Traveler 39	30.5	28.48
September 2020	Pool 36	22.31	Traveler 40	24.06	21.94
September 2020	Pool 37	31.13	Traveler 41	30.06	28.58
September 2020	Pool 38	18.53	Traveler 42	18.18	17.21
October 2020	Pool 39	30.74	Traveler 43	30.43	28.52
October 2020	Pool 40	20.62	Traveler 44	22.65	18.91
October 2020	Pool 41	21.93	Traveler 45	21.5	21.95
October 2020	Pool 42	20.93	Traveler 46		20.3
October 2020		20.93	Traveler 47		19.07

October 2020	Pool 43	37.7	Traveler 48	33.89	33.76
October 2020	Pool 44	16.41	Traveler 49	16.95	16.21
October 2020	Pool 45	18.02	Traveler 50		16.31
October 2020		18.02	Traveler 51		29.09
October 2020	Poo 46	26.15	Traveler 52	24.18	23.05
October 2020	Poo 47	30.62	Traveler 53	29.98	29.13
October 2020	Poo 48	18.27	Traveler 54	18.66	16.39
October 2020	Poo 49	23.16	Traveler 55	21.01	21.52
October 2020	Poo 50	13.95	Traveler 56	11.74	12.86
October 2020	Pool 51	18.65	Traveler 57		18.38
October 2020		18.65	Traveler 58		21.82
October 2020	Pool 52	32.11	Traveler 59	30.08	30.88
October 2020	Pool 53	19.09	Traveler 60	16.52	17.02
October 2020	Pool 54	31.97	Traveler 61	31.33	30.95
October 2020	Pool 55	17.49	Traveler 62	18.1	16.11
October 2020	Pool 56	21.24	Traveler 63	20.21	19.22
October 2020	Pool 57	17.43	Traveler 64	17.01	16.38
October 2020	Pool 58	20.62	Traveler 65		20.64
October 2020	Pool 59	29.02	Traveler 66		28.3
October 2020	Pool 60	29.5	Traveler 67		31.21
October 2020		29.5	Traveler 68		31.1
October 2020	Pool 61	25.15	Traveler 69		23.68
October 2020	Pool 62	22.7	Traveler 70		22.01
October 2020	Pool 63	36.5	Traveler 71		33.14
October 2020	Pool 64	21.4	Traveler 72		21.87
October 2020	Pool 65	37.62	Traveler 73		33.84
October 2020	Pool 66	36.5	Traveler 74		32.75
October 2020	Pool 67	18.44	Traveler 75		15.43
October 2020		18.44	Traveler 76		27.2
October 2020		18.44	Traveler 77		23,00
October 2020		18.44	Traveler 78		22.95
October 2020	Pool 68	22.98	Traveler 79		22.28
October 2020		22.98	Traveler 80		34.6
October 2020		22.98	Traveler 81		25.19
October 2020	Pool 69	19.13	Traveler 82		15.81
October 2020		19.13	Traveler 83		24.23
October 2020	Pool 70	24.74	Traveler 84		23.18
October 2020	Pool 71	24.17	Traveler 85		22.05
October 2020	Pool 72	29.56	Traveler 86		28.18
October 2020	Pool 73	28.02	Traveler 87		27.02
October 2020	Pool 74	28.91	Traveler 88		27.12
October 2020	Pool 75	37.02	Traveler 89		35.1
October 2020	Pool 76	26.09	Traveler 90		24.18
October 2020	Pool 77	26.24	Traveler 91		25.63
October 2020	Pool 78	20.96	Traveler 92		21.26
October 2020		20.96	Traveler 93		20.08
October 2020	Pool 79	28.93	Traveler 94		25.77
October 2020	Pool 80	20.09	Traveler 95		20.93
October 2020	Pool 81	31.04	Traveler 96		28.89
October 2020	Pool 82	21.45	Traveler 97		18.16

October 2020	Pool 83	28.01	Traveler 98		26.84
October 2020	Pool 84	23.4	Traveler 99		25.11
October 2020	Pool 85	16.78	Traveler 100		15.74
October 2020		16.78	Traveler 101		22.87
October 2020	Pool 86	20.01	Traveler 102		18.85
October 2020	Pool 87	34.34	Traveler 103		30.4
October 2020	Pool 88	20.53	Traveler 104		19.29
October 2020	Pool 89	16.28	Traveler 105		12.55
October 2020	Pool 90	21.66	Traveler 106		20.26
October 2020	Pool 91	37.8	Traveler 107		32.57
October 2020	Pool 92	34.08	Traveler 108		26.21
October 2020	Pool 93	19.87	Traveler 109		18.04
October 2020	Pool 94	23.03	Traveler 110		22.82
October 2020	Pool 95	32.64	Traveler 111		30.47
October 2020	Pool 96	19.54	Traveler 112		17.29
October 2020	Pool 97	21.94	Traveler 113		19.31
October 2020	Pool 98	28.95	Traveler 114		29.39
October 2020	Pool 99	30.29	Traveler 115		29.39
October 2020	Pool 100	31.7	Traveler 116		30.46
October 2020	Pool 101	19.9	Traveler 117		17.92
October 2020	Pool 102	25.85	Traveler 118		21.35
October 2020	Pool 103	18.08	Traveler 119		20.06
October 2020		18.08	Traveler 120		18.01
October 2020	Pool 104	29.38	Traveler 121		29.73
October 2020	Pool 105	31.75	Traveler 122		29.91
October 2020	Pool 106	24.05	Traveler 123		20.67
October 2020	Pool 107	34.2	Traveler 124		31.77
October 2020	Pool 108	31.2	Traveler 125		30.37
October 2020	Pool 109	28.62	Traveler 126		26.89
October 2020	Pool 110	17.28	Traveler 127		16.5
October 2020	Pool 111	24.18	Traveler 128		23.49
October 2020	Pool 112	30.68	Traveler 129		28.51
October 2020	Pool 113	17.28	Traveler 130		21.56
October 2020		17.28	Traveler 131		17.12
October 2020	Pool 114	17.73	Traveler 132		29.38
October 2020		17.73	Traveler 133		15.95
October 2020	Pool 115	21.5	Traveler 134		18.27
October 2020	Pool 116	23.81	Traveler 135		23.14
October 2020	Pool 117	14.05	Traveler 136		12.76
October 2020	Pool 118	26.19	Traveler 137		22.96
October 2020	Pool 119	27.58	Traveler 138		26.02
October 2020	Pool 120	19.43	Traveler 139		19.44
October 2020	Pool 121	24.1	Traveler 140		25.03
October 2020	Pool 122	16.76	Traveler 141		16.03
October 2020		16.76	Traveler 142		20.3
October 2020	Pool 123	20.67	Traveler 143		19.71
October 2020	Pool 124	23.08	Traveler 144		23.19
October 2020		23.08	Traveler 145		23.71
October 2020	Pool 125	15.43	Traveler 146		16.7
October 2020	Pool 126	24.29	Traveler 147		22.62

October 2020	Pool 127	20.99	Traveler 148		18.34
October 2020	Pool 128	18.25	Traveler 149		15.87
October 2020	Pool 129	18.53	Traveler 150		16.31
October 2020	Pool 130	31.86	Traveler 151		34.6
October 2020	Pool 131	16.49	Traveler 152		32.6
October 2020		16.49	Traveler 153		17.71
October 2020		16.49	Traveler 154		17.51
October 2020	Pool 132	18.17	Traveler 155		18.42
November 2020	Pool 133	19.06	Traveler 156		19.38
November 2020		19.06	Traveler 157		18.3
November 2020	Pool 134	25.91	Traveler 158		24.8
November 2020	Pool 135	30.52	Traveler 159		19.09
November 2020	Pool 136	26.13	Traveler 160		28.3
November 2020	Pool 137	17.49	Traveler 161		34.05
November 2020		17.49	Traveler 162		14.43
November 2020	Pool 138	32.88	Traveler 163		27.76
November 2020		32.88	Traveler 164		32.35
November 2020	Pool 139	20.46	Traveler 165		18.5
November 2020	Pool 140	25.46	Traveler 166		23.33
November 2020	Pool 141	23.26	Traveler 167		21.24
November 2020	Pool 142	12.89	Traveler 168		17.05
November 2020	Pool 143	26.24	Traveler 169		26.42
November 2020	Pool 144	25.02	Traveler 170		21.35
November 2020	Pool 145	22.38	Traveler 171		18.15
November 2020	Pool 146	21.65	Traveler 172		15.04
November 2020	Pool 147	26.1	Traveler 173		23.25
November 2020	Pool 148	21.8	Traveler 174		16.47
November 2020	Pool 149	24.37	Traveler 175		25.47
November 2020	Pool 150	32.43	Traveler 176		34.55
November 2020	Pool 151	25.03	Traveler 177		23.85
November 2020	Pool 152	12.72	Traveler 178		11.4
November 2020	Pool 153	28.07	Traveler 179		27.11
November 2020	Pool 154	29.03	Traveler 180		25.94
November 2020	Pool 155	30.62	Traveler 181		27.7
November 2020	Pool 156	24.23	Traveler 182		23.81
November 2020	Pool 157	21.96	Traveler 183		22.41
November 2020	Pool 158	28.25	Traveler 184		27.42
November 2020	Pool 159	32.11	Traveler 185		31.11
November 2020	Pool 160	21.04	Traveler 186		19.07
November 2020		21.04	Traveler 187		28.18
November 2020	Pool 161	23.33	Traveler 188		21.06
November 2020	Pool 162	18.21	Traveler 189		17.23
November 2020	Pool 163	15.27	Traveler 190		13.41
November 2020	Pool 164	31.99	Traveler 191		27.56
November 2020	Pool 165	18.02	Traveler 192		18.37
November 2020	Pool 166	23.86	Traveler 193		23.59
November 2020	Pool 167	24.05	Traveler 194		23.01
November 2020	Pool 168	23.69	Traveler 195		21.64
November 2020	Pool 169	27.14	Traveler 196		28.04
November 2020	Pool 170	12.96	Traveler 197		14.77

November 2020	Pool 171	22,00	Traveler 198		19.83
November 2020	Pool 172	27.9	Traveler 199		27.22
November 2020	Pool 173	33.23	Traveler 200		30.84
November 2020	Pool 174	20.85	Traveler 201		17.3
November 2020	Pool 175	20.18	Traveler 202		17.62
November 2020	Pool 176	24,00	Traveler 203		21.9
November 2020	Pool 177	24.02	Traveler 204		32,00
November 2020		24.02	Traveler 205		22.26
November 2020	Pool 178	23.26	Traveler 206		20.35
November 2020	Pool 179	27.67	Traveler 207		25.48
December 2020	Pool 180	24.3	Traveler 208		22.93
December 2020	Pool 181	23.03	Traveler 209		20.33
December 2020	Pool 182	34.06	Traveler 210		31.16
December 2020	Pool 183	20.58	Traveler 211		18.5
December 2020	Pool 184	23.02	Traveler 212		21.53
December 2020	Pool 185	30.7	Traveler 213		27.27
December 2020	Pool 186	25,00	Traveler 214		22.04
December 2020	Pool 187	27.14	Traveler 215		24.48
December 2020	Pool 188	30.45	Traveler 216		28.37
December 2020	Pool 189	19.26	Traveler 217		17.09
December 2020	Pool 190	21.73	Traveler 218		21.14
December 2020	Pool 191	25.13	Traveler 219		22.64
December 2020	Pool 192	24.08	Traveler 220		22.68
December 2020	Pool 193	13.8	Traveler 221		11.4
December 2020	Pool 194	25.1	Traveler 222		23.54
December 2020	Pool 195	25.53	Traveler 223		33.76
December 2020		25.53	Traveler 224		24.84
December 2020	Pool 196	20.39	Traveler 225		18.36
December 2020	Pool 197	20.01	Traveler 226		17.45
December 2020	Pool 198	32.59	Traveler 227		29.44
December 2020	Pool 199	19.43	Traveler 228		17.34
December 2020		19.43	Traveler 229		28.96
December 2020	Pool 200	30.82	Traveler 230		28.6
December 2020	Pool 201	26.64	Traveler 231		25.3
December 2020	Pool 202	28.5	Traveler 232		27.38
December 2020	Pool 203	34.1	Traveler 233		34.34
December 2020	Pool 204	34.8	Traveler 234		33.25
December 2020	Pool 205	28.6	Traveler 235		26.55
December 2020	Pool 206	21.53	Traveler 236		22.66
December 2020		21.53	Traveler 237		20.23
December 2020		21.53	Traveler 238		24.39
December 2020	Pool 207	31.15	Traveler 239		28.11
December 2020	Pool 208	20.6	Traveler 240		17.22
January 2021	Pool 209	25.9	Traveler 241		25.1
January 2021	Pool 210	20.21	Traveler 242		18.61
January 2021	Pool 211	23.05	Traveler 243		20.14
January 2021	Pool 212	24.31	Traveler 244		21.28
January 2021	Pool 213	20.19	Traveler 245		17.01
January 2021	Pool 214	23.89	Traveler 246		20.7
January 2021	Pool 215	25.05	Traveler 247		22.7

January 2021	Pool 216	20.35	Traveler 248		17.78
January 2021	Pool 217	26,00	Traveler 249		23.4
January 2021	Pool 218	24.1	Traveler 250		34.88
January 2021		24.1	Traveler 251		21.23
January 2021	Pool 219	22.57	Traveler 252		19.1
January 2021	Pool 220	26.94	Traveler 253		25.3
January 2021	Pool 221	29.15	Traveler 254		26.9
January 2021	Pool 222	26.37	Traveler 255		22.1
January 2021	Pool 223	31.53	Traveler 256		27.79
January 2021	Pool 224	35.73	Traveler 257		32.86
January 2021	Pool 225	20.05	Traveler 258		17.33
January 2021	Pool 226	28.25	Traveler 259		23.41
January 2021	Pool 227	29.17	Traveler 260		28.42
January 2021	Pool 228	29.16	Traveler 261		28.52
January 2021	Pool 229	21.02	Traveler 262		19.03
January 2021	Pool 230	22.19	Traveler 263		18.48
January 2021	Pool 231	24.35	Traveler 264		21.9
January 2021	Pool 232	27.31	Traveler 265		24.67
January 2021	Pool 233	23.62	Traveler 266		20.46
February 2021	Pool 234	22.91	Traveler 267		20.84
February 2021	Pool 235	20.75	Traveler 268		18.65
February 2021	Pool 236	29.33	Traveler 269		26.87
February 2021	Pool 237	18.38	Traveler 270		17.03
February 2021		18.38	Traveler 271		15.56
February 2021	Pool 238	36.38	Traveler 272		33.02
February 2021	Pool 239	ND	Traveler 273		29.07

ILM: Institut Louis Malardé; ND: not determined
